# Effects of Licorice on Relief and Recurrence of Menopausal Hot Flashes

**Published:** 2012

**Authors:** Fatemeh Nahidi, Elham Zare, Faraz Mojab, Hamid Alavi-majd

**Affiliations:** a *DepartmentofMidwiferyandReproductiveHealth, SchoolofNursingandMidwifery, ShahidBeheshtiUniversityofMedicalSciences, Tehran, Iran*; b *SchoolofPharmacyandPharmaceuticalSciencesResearchCenter, ShahidBeheshtyUniversityofMedicalSciences, Tehran, Iran. *; c *DepartmentofBiostatistics, SchoolofParamedical, ShahidBeheshtiUniversityofMedicalSciences, Tehran, Iran.*

**Keywords:** Menopause, Menopausal women, Vasomotor hot flash, Post menopausal hot flash, Herbal medicine, Licorice

## Abstract

Vasomotor hot flash is the most common and distressful complication of menopausal women. Its treatment is the most frequent clinical challenge. As a result, an effective and harmless therapy is needed. This double-blind controlled clinical trial was conducted to determine the effects of licorice roots on the relief and recurrence of hot flash in menopausal women referring to the healthcare centers affiliated to Shahid Beheshti Medical University in 2010.

Ninety menopausal women complaining of hot flash were selected by reviewing their records in healthcare centers and randomly divided into 2 licorices (3 capsules daily containing 330 mg licorice abstract) and placebo (3 capsules daily containing 330 mg starch) groups over the 8 weeks of intervention and 4 weeks of follow-up. Two weeks prior to the intervention, the severity as well as frequency of hot flashes and the foods taken were asked and documented with questionnaires and data sheets. Data within and between the groups were analyzed by ANOVA with repeated measurements and t-test respectively.

Means of age and body mass index (BMI) of the subjects in licorice and placebo groups were 53 ± 3.2, 52.69 ± 2.8, 24.71 ± 3.2 and 23.61 ± 3.3, respectively. The groups were similar in terms of intervening variables. The frequency of hot flash decreased significantly in the experimental (than the placebo group) and this lasted for 2 weeks after the administration of the capsules. The severity of hot flash decreased in the licorice group as well. This decrease was also seen in the placebo group in the first week of the intervention. Decreased hot flash in the placebo group was only significant after the 1^st^ week of intervention compared to the previous period. Recurrence of frequency and severity of hot flashes occurred 2 weeks after the termination of therapy.

The significant decrease in the placebo group after the 1^st^ week of the intervention may be attributed to the psychological effects of placebo. Licorice roots decreased the frequency and severity of hot flashes. The administration of this harmless, inexpensive herb well accepted by the menopausal women together with the appropriate and continuous physical activities and consumption of dairy products are recommended for relieving this complication.

## Introduction

Hot flash is the primary symptom of menopause and most of the women suffer from it in different levels. It denotes sudden redness of skin, neck and chest with a feeling of hotness in the trunk ending up with severe perspiration (1). Vasomotor hot flash is the most common cause for seeking help in menopausal women (2). Forty percent of premenopausal and 85% of menopausal women complain about vasomotor disorders. It occurs more at nights, leading to arousal (1). Its pathophysiology is unknown; however, the decrease and cessation of estrogen secretion plays a significant role, causing alterations in cerebral mediators and instability of temperature regulation center in hypothalamus (3).

Hot flash and sweating have no inherent risk for health and are not life-threatening. However, they cause discomfort and trouble, affecting daily activities of living (4). Hot flash affects on work, social activities, leisure time, mood, concentration, relationship with others, sexual activities, enjoyment from living and quality of life (5). The most common intervention is hormone replacement therapy (HRT) with many advantages including 70-80% decrease in hot flashes (6). The administration of systemic estrogen is the most effective method to relieve hot flash and sleep disorders resulted from it (2). However, such complications as heart attack, coronary artery diseases, myocardial infarction, thromboembolic events and breast cancer have resulted from it (7).

With respect to the risks of HRT, it should be used at the lowest dose and shortest duration (2). In addition, estrogen is contraindicated in 10% of women; accordingly, HRT is utilized in less than 20% of women (8). In Iran, findings of a study showed that only 8.75% of menopausal women used HRT (9). In recent decades, such alternative therapies as nutrition, exercise, aromatherapy, homeopathy, relaxation and herbal medicine have been increasingly utilized in a surprising manner with an effective relief of menopausal complications (10). Herbal products relieve both physical and psychological symptoms with usual estrogenic effects (11).

Licorice is an herb administered for relieving the menopausal signs and symptoms; however, little is known about its effects (12). Its root is the effective part for the treatment of cough, sore throat and peptic ulcer or as a laxative and antihistaminic agent (13). With respect to the lack of enough evidence regarding the effects of the herb and since it is abundant, cheap and accepted in Iran, this study was conducted to determine the effects of licorice on frequency and severity of hot flashes in menopausal women referring to the healthcare centers affiliated to Shahid Beheshti Medical University in 2010.

## Experimental

The study was a double-blind randomized placebo-controlled clinical trial. Ninety menopausal women referring to the healthcare centers affiliated to Shahid Beheshti Medical University were selected through purposive sampling method as the researchers reviewed medical records of families to find the subjects, their addresses and telephone numbers. The women were then invited by the community health workers to participate in a session and if not, the researchers themselves went to their homes and tried to obtain their written informed consent by introducing themselves and describing the aims and methods of the study.

Then, each subject one by one in turn was assigned to a group to form 2 equal licorice and placebo groups. The subjects were supposed to have the following situations:

Be literate or have a literate person at home; Be between 45-60; Having a body mass index (BMI) below 29; Experiencing amenorrhea for at least 1 year or at most 3 years; Suffering from hot flash and using no drug or hormone to relieve it; Having no known disease in their medical records; Taking no antidepressants or sedatives; Be married or lost their husbands not sooner than 1 year; Experiencing no stressful event (*e.g*. death of close family members, divorce *etc*.) in the past 6 months; Not be vegetarian; Having no allergy to herbs; Having no history of estrogenic cancers in themselves or their close relatives.

The age, duration of amenorrhea frequency and the severity of hot flash as intervening the variables were matched.

Data collection tools included 3 questionnaires, 2 information forms, a scale, a meter band and a sphygmomanometer. Questionnaire 1 included demographic items, menstruation history, gestation history, exercise, frequency as well as the severity of hot flash and coping strategies with it, which was completed before the therapy. Questionnaire 2 contained clients’ activities during the past week including exercise, unpleasant events and coping strategies with hot flash, which was completed by the researchers at the end of each week. Questionnaire 3 assessed hot flash severity based on a visual linear scale (pain ruler), which was asked by the researchers at the end of each week.

Information form 1 was completed by the subjects who had documented their daily food intake 2 weeks before taking the herb until the end of the intervention. Information form 2, related to the frequency as well as the severity of hot flash, was also completed by the subjects 2 weeks before the intervention until 12 weeks after it and was weekly collected. Content method was used for the validity of the tools. For validity of the scale, a standard one was used and for its reliability, it was tested with a 2 Kg standard weight and after each ten measurement, calibrated via the same weight. For height measurement, a nonflexible meter band resisting to temperature changes was used.

Neither the researchers nor the subjects were aware of the content of capsules since the capsules were coded by a pharmacist. Herb capsules contained 330 mg licorice root abstract while placebo capsules had 330 mg starch with the same shape. The subjects took them for 8 weeks 3 times a day (morning, noon and night). To assess recurrence, the subjects were followed up for 4 weeks after the capsule administration. Following the therapy and follow-up stages, data were extracted from the forms and analyzed through ANOVA with repeated measurements for within-group comparisons and t-test for comparisons between the groups with SPSS package.

## Results and Discussion

No significant difference was found between the groups in terms of age, BMI, occupation, occupation of spouse, education, family income, menarche, time of the last menstruation, onset of hot flash, number of live children, cold liquids, daily stress and anger, coping strategies and measures to decrease or relieve hot flash (Table 1).

**Table 1:** Distribution of the subjects in terms of intervening factors in the 2 groups.

**Variable **	**Group**		**Licorice (n = 45)**	**Placebo (n = 45)**
Age			53 ± 3.19	52.69 ± 2.80
BMI			24.71 ± 3.17	23.61 ± 3.30
No. of children			3.4 ± 1.2	3.1 ± 0.4
Age of menarche			13.3 ± 1.1	12.8 ± 1.5
Last menstruation			1.9 ± 0.9	2 ± 1.0
Live children			3.1 ± 1.9	3.5 ± 2.1
Occupation		Household	37 (82.2%)	36 (80%)
		Employed	4 (9.1%)	3 (6.7%)
		Home occupation	4 (8.9%)	6 (13.3%)
Occupation of spouse		Businessman	28 (62.2%)	28 (62.2%)
		Employee	4 (9.1%)	7 (15.4%)
		Worker	7 (15.5%)	7 (15.6%)
		Jobless	6 (13.2%)	3 (6.8%)
Education		Illiterate	4 (8.9%)	2 (4.4%)
		Primary	11 (24.4%)	9 (22.2%)
		Secondary	5 (11.1%)	15 (33%)
		High school or higher	25 (55.5%)	19 (41.4%)
Residence		Private	13 (28.9%)	21 (46.7%)
		Rental	27 (60%)	17 (37.7%)
		Relatives	5 (11.1%)	7 (15.6%)
Family income		< 250,000	15 (33.3%)	13 (28.8%)
		250,000-500,000	14 (31.1%)	16 (35.6%)
		500.000-1000,000	16 (35.6%)	16 (35.6%)
Discomfort and angriness		Always	3 (6.7%)	6 (13.3%)
		Occasionally	39 (86.6%)	35 (77.8%)
		Never	3 (6.7%)	4 (8.9%)
Coping strategies		Fight	2 (4.4%)	1 (2.4%)
		Entertainment	15 (33.3%)	17 (37.7%)
		Coping	19 (42.3%)	16 (35.5%)
		Impatience	9 (20%)	11 (24.4%)
Exercise		Yes	22 (48.9%)	13 (28.3%)
		No	23 (51.1%)	32 (71.7%)
Onset time of hot flash		1-2 year	43 (95.6%)	41(91.2%)
		2-3 year	2 (4.4%)	4 (8.8%)
Cool liquids		Yes	13 (28.9%)	9 (20%)
		No	10 (22.2%)	10 (22.2%)
		Occasionally	22 (48.9%)	26 (57.8%)
Interventions to relieve or decrease		Yes	5 (11.2%)	7 (15.5%)
		No	40 (88.8%)	38 (84.4%)

**Table 2:** Mean frequencies of daily hot flash before the therapy, over 8 weeks and 4 weeks after it in the 2 groups

**Time of treatment**	**Licorice (n = 45)**	**Placebo (n = 45)**	**Test result**
**Group**			
before the therapy	7.70 ± 0.45	7.73 ± 0.4	NS
1^st^ week of therapy	7.46 ± 0.57	7.71 ± 0.32	p < 0.002
2^nd^ week of therapy	7.35 ± 0.45	7.70 ± 0.23	p < 0.001
3^rd^ week of therapy	7.09 ± 0.56	7.71 ± 0.38	p < 0.001
4^th^ week of therapy	6.84 ± 0.74	7.70 ± 0.39	p < 0.001
5^th^ week of therapy	6.79 ± 0.76	7.71 ± 0.49	p < 0.001
6^th^ week of therapy	6.70 ± 0.85	7.74 ± 0.63	p < 0.001
7^th^ week of therapy	6.56 ± 0.92	7.76 ± 0.49	p < 0.002
8^th^ week of therapy	6.43 ± 0.94	7.75 ± 0.55	p < 0.001
1^st^ week of follow up	6.44 ± 0.94	7.77 ± 0.52	p < 0.001
2^nd^ week of follow up	6.48 ± 0.95	7.72 ± 0.36	p < 0.001
3^rd^ week of follow up	7.56 ± 0.99	7.69 ± 0.35	NS
4^th^ week of follow up	7.66 ± 1.02	7.75 ± 0.39	NS
Test result over time	p < 0.001	NS	

Mean frequencies of hot flash in licorice and placebo groups before, during and after the therapy are presented in Table 2. T-test showed no significant difference between the groups before the therapy in terms of the frequencies. Mean frequencies of hot flash reached from 7.70 ± 0.45 before the therapy to 6.43 ± 0.94 at the end of the 8^th^ week and returned to 7.66 ± 1.02 at the 4^th^ week of follow-up. ANOVA with repeated measurements showed significant differences between the mean frequencies before the therapy, the 1^st^ to the 8^th^ week of therapy and the first 2 weeks after it (follow-up period) (p < 0.001); however, no significant difference was found between the frequencies before the therapy and the last 2 weeks of follow-up. In other words, frequencies of daily hot flash significantly decreased over 8 weeks of the therapy and 2 weeks after it compared to those before the intervention. In fact, the effects of phytoestrogen in licorice root remained until 2 weeks after the therapy. In contrast, the mean frequencies in the placebo group before, during and after the intervention showed no significant difference.

**Figure 1:**
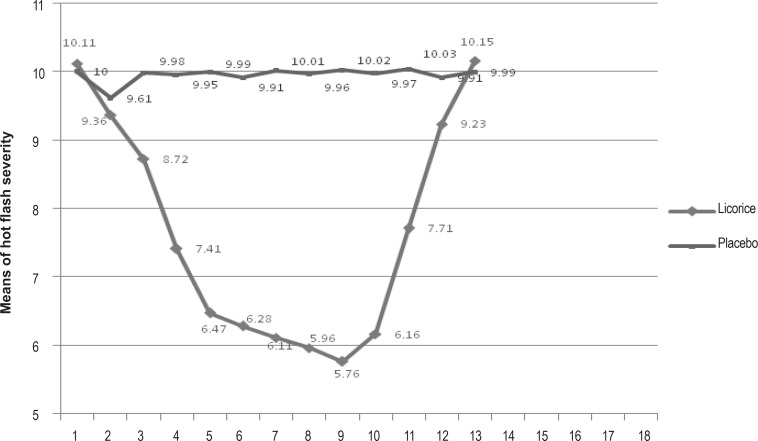
Means of hot flash severity 2 weeks before the therapy, 8 weeks during and 4 weeks after it in 2 groups

Hot flash severity changes before, during and after the therapy are presented in Figure 1. T-test showed no significant difference between the means of hot flash severity in the 2 groups before the therapy. Hot flash severity decreased in both groups from the beginning of the therapy, *i.e.* the means of hot flash severity in licorice and placebo groups were 10.1 ± 1.9 and 10 ± 1.7 before as well as 9.4 ± 2.1 and 9 ± 1.8 in the 1^st^ week of the therapy respectively. T-test showed no significant difference between the groups at this stage (p < 0.002). In addition, the test showed a significant decrease in licorice and in placebo groups before and 1 week after the intervention. However, the decrease was continued until 2 weeks after the therapy in the licorice group while it was only significant in the placebo group in the 1^st^ week of therapy and from the 2^nd^ week to the end of follow-up stage, no significant difference was found compared to the beginning of the study.

Changes of hot flash severity in the subjects of both groups before, during and after (follow-up stage) the therapy is presented in Table 3. As shown, 22.7% of the subjects in the licorice group had experienced severe hot flash before the intervention while it was reduced to 2.3% after the course of therapy. At the end of 4 weeks follow-up, 24.4% of the subjects suffered from severe hot flash. In addition, 31.1% of the subjects in the licorice group experienced mild hot flash while at the end of 8 weeks of therapy, 64.4% of women and, after follow-up, 24.4% of them suffer from it. In the placebo group, 35.5% of the subjects before the therapy and 40% of them after 4 weeks of intervention had mild hot flash; the corresponding figures for severe hot flash were 24.4% and 15.6%.

## Discussion

Findings showed that licorice root is effective in reducing the frequency and severity of hot flash. Comparing the mean frequencies of hot flash in the weeks before the therapy, 8 weeks of therapy and 4 weeks of follow-up through ANOVA with repeated measurements, there was found a significant difference in the licorice group and no difference in the placebo group. In other words, licorice could decrease daily hot flash frequencies during 8 weeks while no decrease was found at the same period in the other group. In addition, the significant difference in the licorice group was seen from the 1^st^ week, increased significantly in time and continued until the 2^nd^ week after the therapy as the recurrence took place from this stage.

**Table 3:** Distribution of the subjects in terms of hot flash severity based on the pain ruler scale before and during the therapy and after the follow-up in both groups

**Groups**	**Hot flash severity**	**Before the treatment (n = 45)**	**During the therapy (n = 45)**	**After the therapy**
	**Time**			
Licorice	Mild	14 (31.1)	29 (64.4)	11 (24.4)
	Moderated	21 (46.6)	15 (33.3)	23 (51.1)
	sever	10 (22.2)	1 (2.3)	11 (24.4)
Placebo	Mild	16 (35.5)	20 (44.4)	18 (40)
	Moderate	18 (40)	15 (33.3)	20 (44.4)
	Sever	11 (24.4)	10 (22.3)	7 (15.6)

Independent t-test showed no significant difference between the mean frequencies of daily hot flash before the therapy in 2 groups, denoting their similarity in this regard. Abbaspour (2003) found that the mean frequencies of 24 h hot flash before and after soya consumption reached from 10.38 to 5.45 in the experimental group and from 10.41 to 9 in the control group during 4 weeks, and the difference became significant from the 3^rd^ week (14). In comparison with our results, it seems that the effects of licorice occur sooner. Besides, our results are congruent with the findings of Jafari *et al*. who found a reduction in hot flash and an improvement in the quality of life through studying the phytoestrogens in 2 derivatives of isoflavone of red clover (*Trifolium pratenes*) (15).

Kazemian *et al.* (2006) studied the effects of phytoestrogen in valerian on hot flash over 2 months. Before and 1 month after as well as before and 2 months after therapy, a significant difference was found in the frequency and severity of hot flash (16). Nahidi *et al.* (2006) indicated that the phytoestrogen in *Pimpinella anisum* is effective on hot flash frequency of menopausal women from the 2^nd^ week of therapy in urban and rural areas of Qazvin (17). Nahas *et al.* (2004) found a complete relief of hot flash in 44%, a relative relief in 26% and no relief in 20%. In placebo group, 12%, 28% and 60% were completely, relatively or indifferently relieved respectively (18). With respect to the long duration of therapy in these 2 studies, it is expected that licorice, if taken longer, may have better effects. Whether licorice can completely relieve, hot flash needs further investigation. However, the effect of it for reducing the hot flash frequency is evident in this study and the assessment of recurrence after therapy has not been reported before.

Comparing the means of hot flash severity before, during and after the therapy through the ANOVA with repeated measurements, indicated the significant and nonsignificant differences in the intervention and placebo groups respectively. In the licorice group, a significant difference was found in hot flash severity from the 1^st^ week and the difference continued to increase significantly until the 2^nd^ week after the therapy as recurrence occurred following the 2^nd^ week of follow-up. In the placebo group, this difference was only significant in the 1^st^ week, which may be attributed to the psychological effects of the intervention. However, the difference did not last since then. In addition, t-test showed no significant difference in hot flash severity between the groups before and in the 1^st^ week of therapy, which indicated the congruity of the groups before the therapy and similarity of estrogenic effects of licorice root as well as psychological effects of placebo in the 1^st^ week of intervention.

In the study of Nahidi *et al.* (2006), the phytoestrogen effect of *Pimpinella anisum* on hot flash severity was well indicated (17). This was also shown by Jafari *et al*. (2003) who studied the effects of 2 derivatives of red clover (*Trifolium pratenes*) on hot flash severity (15). In addition, Hiric and colleagues (2006) found a similar decrease in studying the effects of humulus lupulus on hot flash. Kazemian *et al.* (2006) studied the phytoestrogen effects of valerian on hot flash severity and found a significant difference before and after 1 month as well as before and after 2 months of therapy. They also found a significant decrease in their placebo group (16). The same was found in the study of Nahas *et al.* regarding the useful effects of isoflavone of soya in women prohibited from hormone therapy (18). Albertazi also studied the effects of soya on hot flash and found a 45% decrease in intervention group compared with a 30% decrease in placebo group, denoting the psychological effects of placebo (6). The significant effect of placebo is a constant and remarkable finding of most studies regarding hot flash. The new aspect of the present study is the assessment of recurrence over 4 weeks after the therapy, which occurred after 2 weeks.

No side effect related to licorice was reported by the subjects. Three women in the intervention group reported blotting in the 2^nd^ 4 week of therapy, which was relieved by discontinuation of the capsules. The women were then referred for further check-ups. This may be attributed to the estrogenic effect of licorice root. Since the subjects had no significant difference in terms of duration in hot flash, the length of menopause, frequency as well as severity of hot flash and life style, stressful factors, exercise and diet were controlled before and during the study. To avoid the interfering effects of foods containing phytoestrogens with licorice, the subjects were asked to provide a food diary to control this intervening variable. This can give more confidence in the effects of licorice on frequency and severity of hot flash. Therefore, licorice as an herb containing phytoestrogen can be prescribed for menopausal women suffering from hot flash.

As a result, prior to any intervention for hot flash, consultation with this group is essential to make them familiar with different therapeutic measures including hormonal, chemical and herbal agents. In addition, all advantages and disadvantages of the measures should be provided for them to have the right of choice. With respect to the significant effects of life style changes on health, the administration of licorice should be accompanied with necessary instructions for daily exercise, coping strategies with stress, cool liquids, cold living environment and foods containing phytoestrogen for menopausal women. These can be effectively used to relieve hot flash as it influences on different aspects of physical, psychological, familial and social life.

Blood estrogen levels before, during and after the study were not measured. In addition, the severity of hot flash was assessed only by women’s expressions.

With respect to inadequate evidence regarding licorice and controversies concerning the effects of phytoestrogens in comparison with hormonal drugs, further studies are warranted. Furthermore, measuring the blood estrogen levels can better indicate the effects of phytoestrogen in licorice on hot flashes.
